# Articulating spacers: what are available and how to utilize them?

**DOI:** 10.1186/s42836-023-00167-6

**Published:** 2023-04-10

**Authors:** Zhuo Li, Chi Xu, Jiying Chen

**Affiliations:** 1 School of Medicine, Nankai University, Tianjin, 300071 China; 2 Department of Orthopedics, The First Medical Center, Chinese PLA General Hospital, Beijing, 100853 China; 3 Department of Orthopedics, The Fourth Medical Center, Chinese PLA General Hospital, Beijing, 100048 China

**Keywords:** Periprosthetic joint infection, Arthroplasty, Articulating spacer, Outcomes

## Abstract

Periprosthetic joint infection (PJI) is the most devastating complication following total joint arthroplasty (TJA) and is posing a global healthcare challenge as the demand for TJA mounts. Two-stage exchange arthroplasty with the placement of antibiotic-loaded spacers has been shown to be efficacious against chronic PJI. This study aimed to review the key concepts, types, and outcome evaluations of articulating spacers in the two-stage exchange for PJI. Previous studies indicated that articulating spacers have been widely used due to better functional improvement and a comparable infection control rate relative to static spacers. Several types of articulating spacers are reportedly available, including hand-made spacers, spacers fashioned from molds, commercially preformed spacers, spacers with additional metal or polyethylene elements, new or autoclaved prosthesis, custom-made articulating spacers, and 3D printing-assisted spacers. However, limited evidence suggested no significant difference in clinical outcomes among the different subtypes of articulating spacers. Surgeons should be familiar with different treatment strategies when using various spacers to know which is the most appropriate.

## Introduction

The incidence of periprosthetic infection (PJI) following total knee arthroplasty (TKA) stands somewhere between 0.5% to 2%, and the incidence after total hip arthroplasty (THA) is relatively low, being about 1% [1, 2]. Some studies reported a decreasing trend with the use of modern aseptic techniques [3, 4]. However, with the rapid growth in the number of TKA and THA, the challenge presented by PJI is becoming increasingly severe [5]. Several treatment strategies are currently available for PJI, including one-stage exchange arthroplasty, two-stage exchange arthroplasty, irrigation and debridement, and, in extreme cases, salvage surgery, all of which are associated with a high healthcare cost [6]. Given the high infection control rate, a two-stage revision with the placement of an antibiotic-loaded spacer remains the gold standard for managing chronic PJI [7, 8].

The study of antibiotic-impregnated bone cement began in the early 1970s, reporting that it could reduce the risk of PJI in primary arthroplasty [9]. In 1979, Hovelius *et al.* [10], for the first time, described using gentamicin-loaded cement spheres for infection control in the first-stage hip revision. In 1988, Cohen *et al.* [11] employed an antibiotic-polymethyl methacrylate (PMMA) spacer block to fill the joint cavity after debridement. The initial spacer was static and did not allow for joint movement. Prolonged immobilization and lack of activity can cause bone loss, joint stiffness, and soft tissue contracture, leading to severe complications at reimplantation [12]. The aforementioned issues can be solved by an articulating spacer, which allows for functional movement of the residual joint and provides better conditions for reimplantation [12]. It remains to be noted that articulating spacers should be avoided in cases of severe bone loss or soft tissue defect since they may not offer sufficient stability [13]. Recently, a number of studies have assessed the performance of various types of spacers in the two-stage revision, but high-quality guidelines remain scarce.

This study aimed to provide an instructional review of the key concepts, types, and outcome evaluations of articulating spacers in the two-stage exchange of PJI.

## Antibiotic-loaded cement spacers

**Fig. 1:**
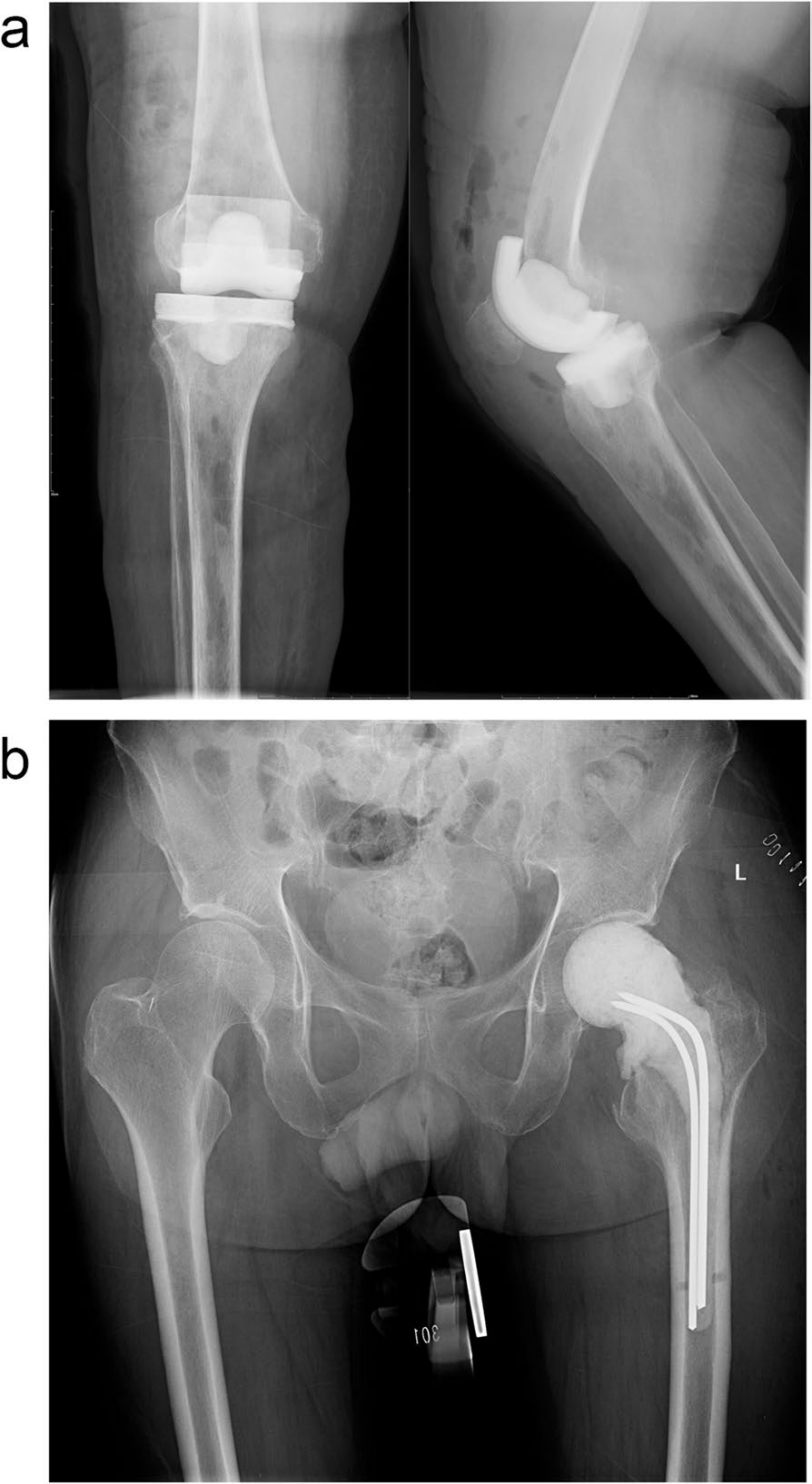
An example of a typical knee (**a**) or hip (**b**) spacer

A typical knee or hip antibiotic-loaded spacer is shown in Fig. 1. Antibiotic-loaded spacers have two primary roles in two-stage exchange arthroplasty: filling the joint cavity, which tensions the soft tissues while restoring limb length, and antibiotic elution. The spacer could fill the dead space inside the joint after the removal of the prosthesis and debridement, which contributes to the maintenance of soft tissue tone and bone quality [14]. Additionally, high intra-articular antibiotic concentrations have been proven available for topical administration via spacers, enabling control of bacterial burden [15]. To effectively eliminate pathogens in biofilms, the antibiotic-loaded spacer is almost the only way to go, as it does not simultaneously increase the concentration of antibiotics in the blood or urine [14, 16]. Historically, the safety of cement spacers has been widely reported, with only a few cases reports describing toxic complications regarding spacers [17, 18].

The antibiotics loaded in the spacers should be water-soluble and resistant to high temperatures to maintain their chemical stability [15]. Aminoglycoside and glycopeptide antibiotics are the most commonly recommended [19]. Spacers can contain multiple antibiotics to broaden their antimicrobial spectrum. At the same time, single antibiotic impregnation is also optional when it is identified as a pathogen-sensitive antibiotic [20]. Another common issue is the dosage of antibiotics, which is related to several factors, including the drug-eluting properties and the bone cement’s mechanical strength [20, 21]. In general, antibiotics impregnated in spacers for two-stage revision should be of high dose (>3.6 g/40 g PMMA), but excessively high dosage (>8 g/40 g PMMA, or more than 10–15 % of total cement mass) should be avoided [14, 21, 22]. Some studies recommended 3 g vancomycin and 3.6 g tobramycin per bag of cement [22].

The mixing procedures exert an impact on the elution of antibiotics. Compared to mixing in the air, vacuum mixing increases the mechanical strength and decreases the porosity of the cement. Alternatively, if the spacers are hand-made, uneven mixing of the ingredients may affect the elution of the antibiotics [20]. The bone cement and the antibiotic powder should be mixed thoroughly and evenly before mixing into the liquid. The spacer should be placed under pressure-free conditions to avoid a strong adhesion between the spacer and the bone. A standardized procedure facilitates spacer removal at reimplantation [16, 20].

## Types of articulating spacers

### Hand-made spacers

Experienced surgeons can hand-make spacers intraoperatively by using antibiotic-impregnated PMMA. These spacers mimic joint anatomy or kinematics (typical ball and socket joint) [23, 24]. The apparent advantages of this spacer are the lower cost and the ability to be individually constructed to fit specific anatomical configurations. However, it can be time-consuming and carries a high risk of fracture. To address this issue and to facilitate re-removal, "endoskeletons", such as Steinman pins, Kirschner wire, or other similar implants are often placed inside the spacer [25, 26]. Another shortcoming of hand-made spacers is the mismatch with the articulating surfaces, which may lead to instability and dislocation [27]. A spacer 2–3 mm smaller than the acetabulum in the hip PJI is recommended to help keep the spacer in place [28]. In addition, hand-made spacers can be loaded with more pathogen-sensitive antibiotics, which facilitates the eradication of infection in case of positive preoperative cultures, but this requires a balance with the mechanical properties of the spacer [29, 30]. Overall, this technique needs to be honed to make the technique clinically applicable to patients.

### Spacers fashioned from molds

**Fig. 2:**
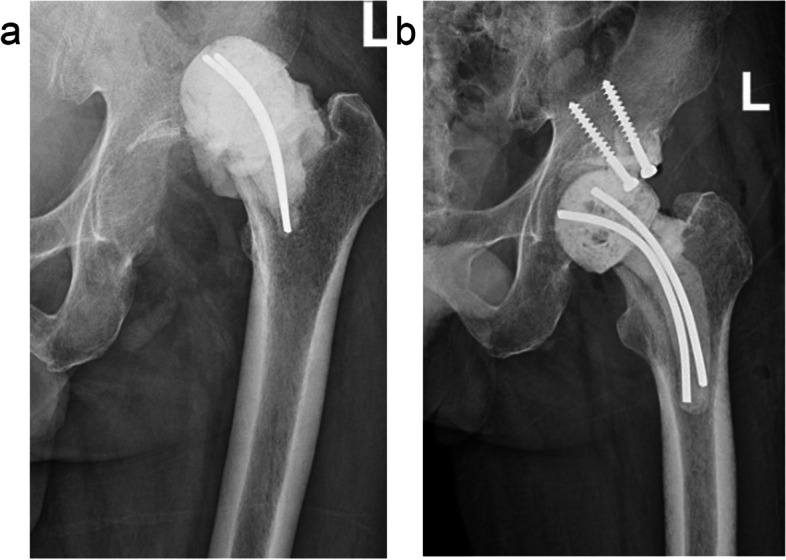
Postoperative dislocation of a hand-made spacer (**a**); another molded spacer was used for spacer exchange and cemented screws were placed to manage the acetabular bone defect and increase hip stability (**b**)

Spacers can also be made from homemade or commercially available molds. These spacers have a more consistent geometry and are not dependent on the surgeon for their construction. The molds are often made of silicone or stainless steel [21, 31, 32]. They are available in different sizes and can be selected intraoperatively based on a comparison against the removed prosthesis and bone anatomy. However, there is no uniformity in the size of the molds, either based on fixed increments [31] or standard prosthetic components [21, 33]. Ha *et al.* [34] described the intraoperative sterilization of the removed femoral components and polyethylene inserts, followed by the construction of a mold from the removed components using lubricant and cement. Like hand-made spacers, spacers fashioned from molds can be personalized with sensitive antibiotics, and there exists a metal endoskeleton to enhance mechanical strength in many cases [21, 35]. In clinical practice, molded spacers can also be used in some specific cases, as appropriate, such as combined acetabular cement screws, to manage bone defects (Fig. 2). Nevertheless, it is noteworthy that molded spacers reportedly increase the cost of the procedure, and their incidence of mechanical complications [13, 35].

### Commercial preformed spacers

So far, a couple of preformed spacers are commercially available, such as the Tecres Spacer-G/K temporary hip/knee spacer (Tecres Spa, Sommacampagna, Verona, Italy). The most significant advantage of preformed spacers is their easy intraoperative use, which saves operative time. Preformed spacers possess more stable mechanical properties and may reduce fracture risk compared to their hand-made counterparts. The antibiotics appear to be released more evenly in the joint as the antibiotics and cement have been uniformly pre-mixed [36]. However, their relatively low antibiotic doses and restrictions on the type of antibiotic may affect their anti-infection efficacy [27, 28, 37, 38]. For instance, Spacer-K pre-mixed with gentamicin has three different sizes with antibiotic doses of 0.8–1.7 g, which are not up to the recommended dose (>3.6 g per 40 g cement) [39]. Minelli *et al.* [40] proposed a strategy to increase the dose of antibiotics in the spacer by drilling holes in the pre-fabricated spacer and filling them with vancomycin-impregnated cement. They concluded that this technique does not adversely affect the release kinetics of antibiotics.

### Spacers with additional metal or polyethylene articulating elements

Implants designed for short-term use and with increased PMMA volume are developed for septic revision, the best known of which is PROSTALAC (prosthesis of antibiotic-loaded acrylic cement, DePuy Synthes, Warsaw, IN, USA). PROSTALAC was initially developed for septic hip revisions and has since been applied to knee [41, 42]. Typically, the system consists of a metal femoral component, a post-stabilized polyethylene tibial component, or a polyethylene acetabular liner [43]. A significant advantage is that it is designed to be semi-constrained to lower the risk of dislocation. Structurally and functionally comparable to the traditional prosthesis, it may be a cost-effective temporary spacer that can reduce the complexity of the procedure [43, 44]. In a 10- to 15-year follow-up study, 99 PJI patients using the PROSTALAC hip spacer attained an 89% long-term treatment success rate, demonstrating that it is a reliable and durable solution [45]. PROSTALAC is also promoted for complicated cases such as total femoral replacement with severe bone loss [46] or reconstruction of infected interprosthetic femoral stem fracture [47]. However, the availability of PROSTALAC is limited, and it is currently not approved for use in many countries.

### New or autoclaved prosthesis

An articulating spacer with a metal-on-polyethylene interface was proposed by Hoffman *et al.* in 1995 [48]. It allows the infected femoral component to be cleaned, autoclaved, and then reimplanted with a new polyethylene liner to treat the infected TKA. After the fixation of these components with antibiotic-impregnated cement, none of the 26 patients developed reinfection. Although this technique substantially reduces the direct cost of constructing a spacer [49], it goes against recommendations of the Food and Drug Administration (USA) and the Medicines and Healthcare Products Regulatory Agency (UK) [13]. To date, several studies have further confirmed the effectiveness of similar techniques [50–54]. *In vitro* and *in vivo* studies have demonstrated the sterility of autoclaved prostheses [55, 56]. It is imperative that the surgeon should remove all periprosthetic tissues and cement before autoclaving [55, 56]. Rigid reusable sterilization containers are preferable and should be close to the operating room for easy delivery [55]. If spore testing is impossible, the prosthesis should receive full-cycle steam sterilization [55]. Similar technique was also employed in septic hip revisions. Hoffman *et al.* [57] autoclaved and reimplanted the infected femoral stem, while Evans *et al.* [58] used a new femoral prosthesis. However, caution should be exercised in interpreting these results, as autoclaving old prostheses is not allowed in many, if not most, hospitals. In this context, the use of a new prosthesis is a more normative and ethical option.

**Fig. 3:**
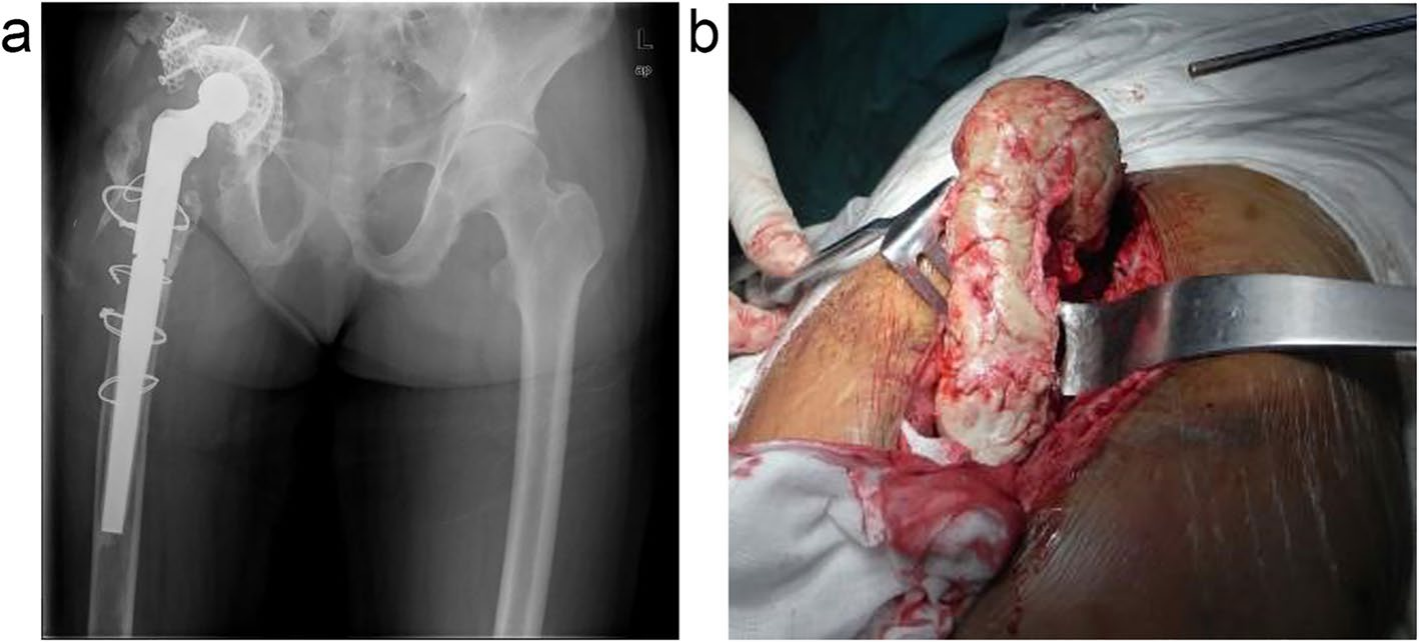
A PJI case with a modular femoral stem. The femoral stem was firmly fixed and difficult to remove. After the removal of the proximal component, a spacer was placed. Pre- (**a**) and intraoperative (**b**) images are presented

**Fig. 4:**
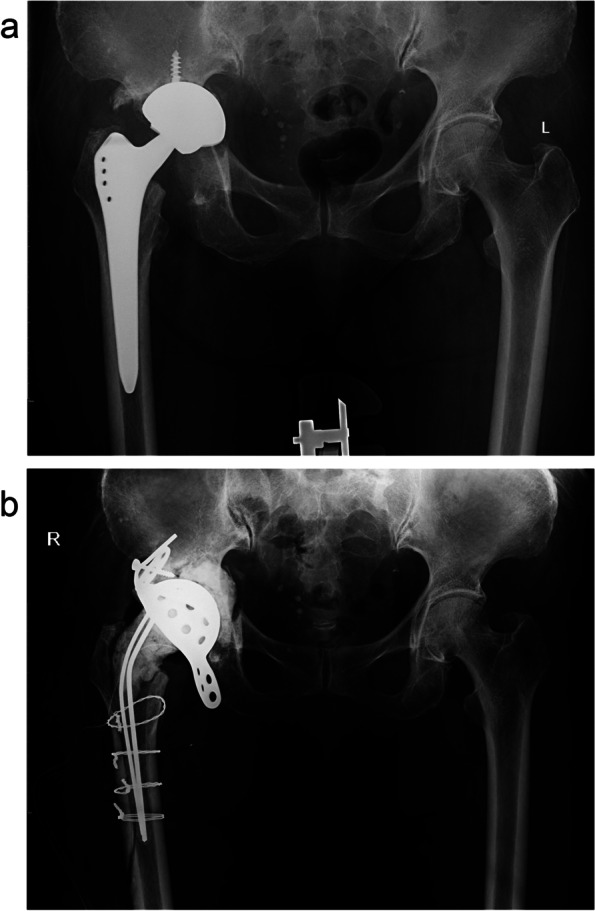
A case of PJI with acetabular protrusion (**a**). Combination of a spacer and a cage to prevent central dislocation of the hip (**b**)

**Fig. 5:**
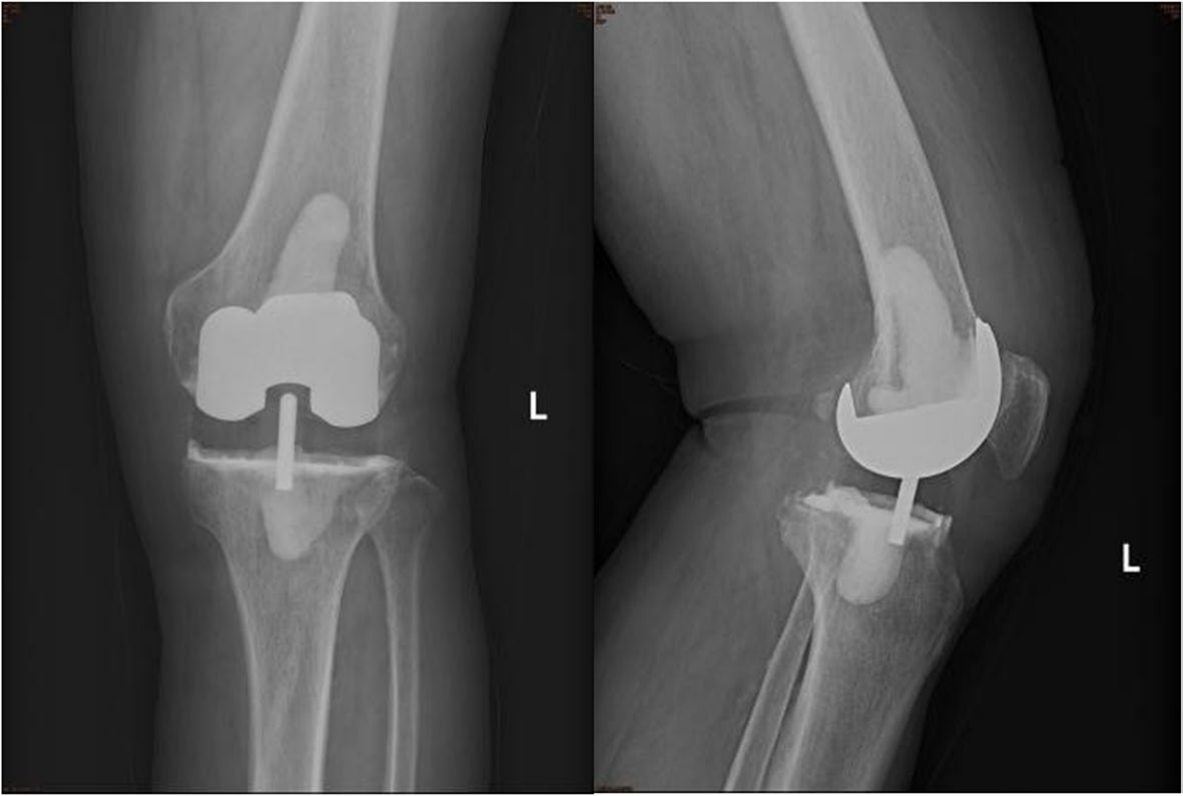
The new prosthesis was combined with high-concentration bone cement for a 1.5-stage exchange, using a Press Fit Condylar femoral component (PFC, DePuy Synthes, Warsaw, IN, USA) and a constrained polyethylene insert

This technology offers great flexibility in two-stage exchange. It allows for partial removal of the prosthesis (Fig. 3) or use in combination with other tools to manage complications (Fig. 4). In addition, it gives the opportunity to perform a 1.5-stage exchange arthroplasty as a substitute for traditional two-stage exchange to manage PJI (Fig. 5). In a 1.5-stage exchange, the infected knee is resected, and an articulating spacer is placed with the intent to stay *in situ* as long as the patient can tolerate it [59]. Theoretically, it is advantageous in that it potentially avoids a second operation. Hernandez *et al*. evaluated 27 patients who underwent 1.5-stage exchange, and only three developed recurrent infections during a 2.7-year follow-up [59]. Nabet *et al.* [60] further found that postoperative complications were lower among 1.5-stage exchanges compared to two-stage exchanges.

### Custom-made articulating spacers (CUMARS)

CUMARS was developed in 2001, and this spacer system includes the Exeter Universal Femoral stem (Stryker, Mahwah, NJ, USA) and a polyethylene acetabular liner [61]. It is comparable, in treatment principles, to PROSTALAC, but its components are more common and readily available [61]. They provide better joint function during the pre-reimplantation period, allowing for full weight-bearing of the lower extremity. More recently, CUMARS was extensively reported to be associated with better inter-stage functionality, easier removal, and excellent infection control [62–65]. Moreover, for low-demand patients with severe comorbidities, a large volume of antibiotic-impregnated cement can be used to hold the spacer in place as firmly as possible [61]. These so-called "long-term spacers" may obviate the need for reimplantation, a potential approach to 1.5-stage exchange arthroplasty [61, 63]. Besides, Quayle *et al.* [62] described use of a modified CUMARS with a long femoral stem to treat PJI with severe bone loss. However, due to the high cost, further evaluation of the cost-benefit of CUMARS is needed. Recently, Craig *et al.* [13] recommended using CUMARS as an articulated spacer whenever possible: a knee cemented femoral component and a polyethylene tibial component, or a cemented, polished, tapered femoral stem with cemented socket (kiwi procedure).

### 3D printing-assisted articulating spacers

3D printing technology allows for rapid prototyping based on each individual’s unique anatomical configuration, thereby improving the match between components and joints. Previous evaluation of 3D models of spacers by using an "*ad hoc*" virtual planning simulator revealed a strong correlation between the geometric characteristics of the spacer and clinical improvement [66]. In recent years, 3D printing-based spacers have been proposed to enhance joint function and stability [67]. Tsai *et al.* [68] suggested that 3D models with high geometric consistency could be obtained by analyzing the removed femoral and tibial prostheses through reverse engineering techniques. The original models were then scaled up while maintaining the geometry to yield femoral and tibial models of different sizes. The infection eradication rate in their study was 87.5 % (28/32) and mechanical complications occurred in two cases, demonstrating a good prospect of computer-aided design and manufacturing of spacers. Kong *et al.* [67] analyzed CT images of the patient’s contralateral knee to determine the spacer’s size and design the mold. They also modified the structure of the anterior condyle, diseased carriage, tibial column, and tibial platform sliding interface. This technique improved the quality and function of the spacer, increased stability, and reduced dead space inside the joint. What is more, Kim *et al.* [69] explored the use of 3D-printed polylactic acid (PLA) to construct spacers. PLA spacers have superior mechanical properties to PMMA and can elute antibiotics in a controlled manner. Despite the unlimited potential, we must be aware that the above-mentioned studies are either small-scale or still confined to *in vitro* experimentation. The lack of relevant medical regulations and consensus is another limitation.

### Modification of spacers

The femoral component for hip spacers should utilize a metal scaffold to reduce the risk of spacer fracture. For example, when an extended trochanteric osteotomy (ETO) is performed, a Steinmann Pin fixed to the bottom of a cemented femoral stem coated with cement creates a construct that bypasses the distal-most extent of the osteotomy. Bone loss in infected revisions is challenging, and small-to-medium-size defects can be managed with a variety of techniques. The application of screws and cement acetabular augmentation can improve acetabular coverage and mitigate the risk of mechanical failure [70]. Additionally, a large-diameter highly cross-linked polyethylene liner with a backside roughened with a burr may be cemented into place. This technique is useful in lowering the postoperative dislocation rate [71]. As for severe bone loss in an infected knee, a static spacer is preferred. Components with augments or stems are available for reimplantation to further enhance joint stability [72]. No consensus has been reached on the optimal design of the spacer, and more investigations are warranted to meet the needs in varying clinical scenarios.

## Comparison of clinical outcomes

Practically all studies investigating antibiotic-loaded spacers in two-stage exchange revisions have shown similar infection eradication rates using static and articulating spacers, in both the hip and knee [21, 73–76]. Studies on different types of hip spacers have indicated that articulating spacers improved joint function more significantly; nevertheless, some failed to show differences in functional scores [13, 28]. A recent multicenter randomized trial exhibited similar results for static and articulating spacers in the treatment of THA PJI. Still, static spacers were significantly associated with extended length of hospital stay, which may place a substantial financial burden [77]. As for the TKA PJI, several systematic reviews noted that functional scores after reimplantation were similar in both groups but that using an articulating spacer resulted in a better range of motion (ROM) [78–80]. Another recent randomized controlled trial demonstrated that articulating spacers provided higher knee association scores, greater ROM, and shorter hospital stay [12]. Besides, comparative studies have shown that articulating spacers facilitated the preservation of the remaining bone mass, but spacer-related mechanical complications are more common [80, 81]. However, we should be aware of the selection bias between static and articulating spacers in the interpretation of these results: The former can be used in more complicated clinical situations, especially when there exist accompanying severe soft tissue defects. We should concede that static spacers may be favoured over articulating spacers in the cases with poor soft tissue envelope, bone loss, or abductor deficiency [82].

Studies comparing the clinical outcomes using specific subtypes of articulating spacers are scanty. DeBoer *et al.* [83] found comparable efficacy between injection molded and prefabricated articulating spacers. In a systematic review [84] comparing 394 patients receiving autoclaved components and 173 patients with spacers made from new components, Spinarelli *et al.* showed that reinfection rates were similar in both groups, and postoperative ROM was greater in patients with autoclaved components. Another recent study revealed that real-component spacers significantly improved patient comfort compared to all-cement articulating ones [85]. Citak *et al.* [37] analyzed 1631 infected THAs and revealed that preformed articulating spacers were not superior to hand-made spacers in terms of functional outcomes and infection eradication. However, preformed articulated spacers reduced the risk of spacer fracture. Veltman *et al.* [73] reviewed 25 hip studies and found similar infection eradication rates among custom spacers (95 %), prefabricated spacers (96 %), and functional articulating spacers (93 %). In another study concerning TKA PJI, Nodzo *et al.* [6] found no difference in surgical success among homemade molds, autoclaved femoral components, or prefabricated spacers, with the cost being the lowest when molds were used. On the basis of different classification methods, a systematic review indicated that spacers containing bio-inert materials and all-cement spacers had similar infection control rates [86]. Another study by Spivey *et al.* [87] demonstrated increased temporary ROM of metal-on-polyethylene spacers and fewer spacer-related complications. However, we must be aware that multiple factors might confound these results, including surgical technique, case complexity, antibiotic administration, *etc*. Besides, it should be noted that with all articulating spacers in which cement articulates with bone, there is a risk of acetabular bone loss. A retrospective study found progressive acetabular bone loss in 43% of patients following explant and spacer placement [88].

## Conclusions

Two-stage exchange arthroplasty with the placement of antibiotic-loaded spacers has been performed successfully in chronic PJI. Many types of spacers are available for clinical selection, but there is still no definitive evidence of the optimal approach. Articulating spacers appear to provide better functional improvement compared to their static counterparts with a similar rate of infection eradication. Limited evidence suggested no significant difference in clinical outcomes among the different subtypes of articulating spacers. Surgeons should be familiar with the treatment strategies using various spacers to decide which is the most appropriate.

On the basis of our practice, we are inclined to advocate dynamic spacers. We only choose static spacers when knee stabilization is difficult to achieve, for instance, when there are severe soft tissue deficiencies or substantial bone loss. Dynamic spacers are generally used in the hip joint unless multiple spacers have been placed and failed to eradicate the infection. Spacers fashioned from molds are more frequently employed due to their reliable function and cost-effectiveness. In complicated cases, 3D-assisted printed spacers and new prostheses in the 1.5-stage exchange can provide more individualized and flexible treatment options.

## Data Availability

Not applicable.

## Abbreviations

PJI: Periprosthetic joint infection
TJA: Total joint arthroplasty
TKA: Total knee arthroplasty
THA: Total hip arthroplasty
PMMA: Antibiotic-polymethyl methacrylate
CUMARS: Custom-made articulating spacers
PLA: Polylactic acid
ROM: Range of motion

## References

[1] Namba RS, Inacio MC, Paxton EW (2013). Risk factors associated with deep surgical site infections after primary total knee arthroplasty: an analysis of 56,216 knees. J Bone Joint Surg Am.

[2] Edwards JR, Peterson KD, Mu Y, Banerjee S, Allen-Bridson K, Morrell G, Dudeck MA, Pollock DA, Horan TC (2009). National Healthcare Safety Network (NHSN) report: data summary for 2006 through 2008, issued December 2009. Am J Infect Control.

[3] Runner RP, Mener A, Roberson JR, Bradbury TL, Guild GN, Boden SD, Erens GA (2019). Prosthetic Joint Infection Trends at a Dedicated Orthopaedics Specialty Hospital. Adv Orthop.

[4] Wang FD, Wang YP, Chen CF, Chen HP (2018). The incidence rate, trend and microbiological aetiology of prosthetic joint infection after total knee arthroplasty: A 13 years’ experience from a tertiary medical center in Taiwan. J Microbiol Immunol Infect.

[5] Kurtz S, Ong K, Lau E, Mowat F, Halpern M (2007). Projections of primary and revision hip and knee arthroplasty in the United States from 2005 to 2030. J Bone Joint Surg Am.

[6] Nodzo SR, Boyle KK, Spiro S, Nocon AA, Miller AO, Westrich GH (2017). Success rates, characteristics, and costs of articulating antibiotic spacers for total knee periprosthetic joint infection. Knee.

[7] Porrino J, Wang A, Moats A, Mulcahy H, Kani K (2020). Prosthetic joint infections: diagnosis, management, and complications of the two-stage replacement arthroplasty. Skeletal Radiol.

[8] Otto-Lambertz C, Yagdiran A, Wallscheid F, Eysel P, Jung N (2017). Periprosthetic Infection in Joint Replacement. Dtsch Arztebl Int.

[9] Hessert GR, Ruckdeschel G (1970). Antibiotic effects of mixtures of polymethlmethacrylate with antibiotics. Arch Orthop Unfallchir.

[10] Hovelius L, Josefsson G (1979). An alternative method for exchange operation of infected arthroplasty. Acta Orthop Scand.

[11] Cohen JC, Hozack WJ, Cuckler JM, Booth RE (1988). Two-stage reimplantation of septic total knee arthroplasty. Report of three cases using an antibiotic-PMMA spacer block. J Arthroplasty.

[12] Nahhas CR, Chalmers PN, Parvizi J, Sporer SM, Berend KR, Moric M, Chen AF, Austin MS, Deirmengian GK, Morris MJ (2020). A Randomized Trial of Static and Articulating Spacers for the Treatment of Infection Following Total Knee Arthroplasty. J Bone Joint Surg Am.

[13] Craig A, King SW, van Duren BH, Veysi VT, Jain S, Palan J (2022). Articular spacers in two-stage revision arthroplasty for prosthetic joint infection of the hip and the knee. EFORT Open Rev.

[14] Tande AJ, Patel R (2014). Prosthetic joint infection. Clin Microbiol Rev.

[15] van de Belt H, Neut D, Schenk W, van Horn JR, van Der Mei HC, Busscher HJ (2001). Staphylococcus aureus biofilm formation on different gentamicin-loaded polymethylmethacrylate bone cements. Biomaterials.

[16] Lachiewicz PF, Wellman SS, Peterson JR (2020). Antibiotic Cement Spacers for Infected Total Knee Arthroplasties. J Am Acad Orthop Surg.

[17] Duncan CP, Masri BA (1995). The role of antibiotic-loaded cement in the treatment of an infection after a hip replacement. Instr Course Lect.

[18] van Raaij TM, Visser LE, Vulto AG, Verhaar JA (2002). Acute renal failure after local gentamicin treatment in an infected total knee arthroplasty. J Arthroplasty.

[19] Iarikov D, Demian H, Rubin D, Alexander J, Nambiar S (2012). Choice and doses of antibacterial agents for cement spacers in treatment of prosthetic joint infections: review of published studies. Clin Infect Dis.

[20] Samelis PV, Papagrigorakis E, Sameli E, Mavrogenis A, Savvidou O, Koulouvaris P (2022). Current Concepts on the Application, Pharmacokinetics and Complications of Antibiotic-Loaded Cement Spacers in the Treatment of Prosthetic Joint Infections. Cureus.

[21] Hsieh PH, Chen LH, Chen CH, Lee MS, Yang WE, Shih CH (2004). Two-stage revision hip arthroplasty for infection with a custom-made, antibiotic-loaded, cement prosthesis as an interim spacer. J Trauma.

[22] Shahpari O, Mousavian A, Elahpour N, Malahias MA, Ebrahimzadeh MH, Moradi A (2020). The Use of Antibiotic Impregnated Cement Spacers in the Treatment of Infected Total Joint Replacement: Challenges and Achievements. Arch Bone Jt Surg.

[23] Shaikh AA, Ha CW, Park YG, Park YB (2014). Two-stage approach to primary TKA in infected arthritic knees using intraoperatively molded articulating cement spacers. Clin Orthop Relat Res.

[24] MacAvoy MC, Ries MD (2005). The ball and socket articulating spacer for infected total knee arthroplasty. J Arthroplasty.

[25] Faschingbauer M, Reichel H, Bieger R, Kappe T (2015). Mechanical complications with one hundred and thirty eight (antibiotic-laden) cement spacers in the treatment of periprosthetic infection after total hip arthroplasty. Int Orthop.

[26] Peng KT, Kuo LT, Hsu WH, Huang TW, Tsai YH (2011). The effect of endoskeleton on antibiotic impregnated cement spacer for treating deep hip infection. BMC Musculoskelet Disord.

[27] Charette RS, Melnic CM (2018). Two-Stage Revision Arthroplasty for the Treatment of Prosthetic Joint Infection. Curr Rev Musculoskelet Med.

[28] Jacobs C, Christensen CP, Berend ME (2009). Static and mobile antibiotic-impregnated cement spacers for the management of prosthetic joint infection. J Am Acad Orthop Surg.

[29] Jiranek WA, Hanssen AD, Greenwald AS (2006). Antibiotic-loaded bone cement for infection prophylaxis in total joint replacement. J Bone Joint Surg Am.

[30] Lautenschlager EP, Jacobs JJ, Marshall GW, Meyer PR (1976). Mechanical properties of bone cements containing large doses of antibiotic powders. J Biomed Mater Res.

[31] Van Thiel GS, Berend KR, Klein GR, Gordon AC, Lombardi AV, Della Valle CJ (2011). Intraoperative molds to create an articulating spacer for the infected knee arthroplasty. Clin Orthop Relat Res.

[32] Durbhakula SM, Czajka J, Fuchs MD, Uhl RL (2004). Antibiotic-loaded articulating cement spacer in the 2-stage exchange of infected total knee arthroplasty. J Arthroplasty.

[33] Hsu YC, Cheng HC, Ng TP, Chiu KY (2007). Antibiotic-loaded cement articulating spacer for 2-stage reimplantation in infected total knee arthroplasty: a simple and economic method. J Arthroplasty.

[34] Ha CW (2006). A technique for intraoperative construction of antibiotic spacers. Clin Orthop Relat Res.

[35] Duensing IM, Kim BI, Charalambous LT, Case A, Surace PA, Seyler TM, Wellman SS (2022). Clinical Outcomes After Stage-One Antibiotic Coated Molded Hip Spacer. J Arthroplasty.

[36] Anagnostakos K (2017). Therapeutic Use of Antibiotic-loaded Bone Cement in the Treatment of Hip and Knee Joint Infections. J Bone Jt Infect.

[37] Citak M, Masri BA, Springer B, Argenson JN, Kendoff DO (2015). Are Preformed Articulating Spacers Superior To Surgeon-Made Articulating Spacers in the Treatment Of PJI in THA? A Literature Review. Open Orthop J.

[38] Goltzer O, McLaren A, Overstreet D, Galli C, McLemore R (2015). Antimicrobial Release From Prefabricated Spacers Is Variable and the Dose Is Low. Clin Orthop Relat Res.

[39] Lu J, Han J, Zhang C, Yang Y, Yao Z (2017). Infection after total knee arthroplasty and its gold standard surgical treatment: Spacers used in two-stage revision arthroplasty. Intractable Rare Dis Res.

[40] Bertazzoni Minelli E, Benini A, Magnan B, Bartolozzi P (2004). Release of gentamicin and vancomycin from temporary human hip spacers in two-stage revision of infected arthroplasty. J Antimicrob Chemother.

[41] Duncan CP, Beauchamp C (1993). A temporary antibiotic-loaded joint replacement system for management of complex infections involving the hip. Orthop Clin North Am.

[42] Meek RM, Dunlop D, Garbuz DS, McGraw R, Greidanus NV, Masri BA (2004). Patient satisfaction and functional status after aseptic versus septic revision total knee arthroplasty using the PROSTALAC articulating spacer. J Arthroplasty.

[43] Gee R, Munk PL, Keogh C, Nicolaou S, Masri B, Marchinkow LO, Ellis J, Chan LP (2003). Radiography of the PROSTALAC (prosthesis with antibiotic-loaded acrylic cement) orthopedic implant. AJR Am J Roentgenol.

[44] Masri BA, Kendall RW, Duncan CP, Beauchamp CP, McGraw RW, Bora B (1994). Two-stage exchange arthroplasty using a functional antibiotic-loaded spacer in the treatment of the infected knee replacement: the Vancouver experience. Semin Arthroplasty.

[45] Biring GS, Kostamo T, Garbuz DS, Masri BA, Duncan CP (2009). Two-stage revision arthroplasty of the hip for infection using an interim articulated Prostalac hip spacer: a 10- to 15-year follow-up study. J Bone Joint Surg Br.

[46] Arac SS, Boya H (2016). A novel hybrid type (custom-made plus off-the-shelf) total femoral PROSTALAC. Hip Int.

[47] Kamath AF, Austin D, Lee GC (2012). Mating of a PROSTALAC spacer with an intramedullary nail for reconstruction of an infected interprosthetic femoral shaft fracture: a case report. J Orthop Surg (Hong Kong).

[48] Hofmann AA, Kane KR, Tkach TK, Plaster RL, Camargo MP (1995). Treatment of infected total knee arthroplasty using an articulating spacer. Clin Orthop Relat Res.

[49] Kalore NV, Maheshwari A, Sharma A, Cheng E, Gioe TJ (2012). Is there a preferred articulating spacer technique for infected knee arthroplasty? A preliminary study. Clin Orthop Relat Res.

[50] Pietsch M, Hofmann S, Wenisch C (2006). Treatment of deep infection of total knee arthroplasty using a two-stage procedure. Oper Orthop Traumatol.

[51] Cuckler JM (2005). The infected total knee: management options. J Arthroplasty.

[52] Jamsen E, Sheng P, Halonen P, Lehto MU, Moilanen T, Pajamaki J, Puolakka T, Konttinen YT (2006). Spacer prostheses in two-stage revision of infected knee arthroplasty. Int Orthop.

[53] Lee BJ, Kyung HS, Yoon SD (2015). Two-Stage Revision for Infected Total Knee Arthroplasty: Based on Autoclaving the Recycled Femoral Component and Intraoperative Molding Using Antibiotic-Impregnated Cement on the Tibial Side. Clin Orthop Surg.

[54] Kim YS, Bae KC, Cho CH, Lee KJ, Sohn ES, Kim BS (2013). Two-stage revision using a modified articulating spacer in infected total knee arthroplasty. Knee Surg Relat Res.

[55] Lyons ST, Wright CA, Krute CN, Rivera FE, Carroll RK, Shaw LN (2016). Confirming Sterility of an Autoclaved Infected Femoral Component for Use in an Articulated Antibiotic Knee Spacer: A Pilot Study. J Arthroplasty.

[56] Park HJ, Kim HJ, Kim S, Kim SM, Mun JU, Kim J, Kyung HS (2018). Safety of Temporary Use of Recycled Autoclaved Femoral Components in Infected Total Knee Arthroplasty: Confirming Sterility Using a Sonication Method. Clin Orthop Surg.

[57] Hofmann AA, Goldberg TD, Tanner AM, Cook TM (2005). Ten-year experience using an articulating antibiotic cement hip spacer for the treatment of chronically infected total hip. J Arthroplasty.

[58] Evans RP (2004). Successful treatment of total hip and knee infection with articulating antibiotic components: a modified treatment method. Clin Orthop Relat Res.

[59] Hernandez NM, Buchanan MW, Seyler TM, Wellman SS, Seidelman J, Jiranek WA (2021). 1.5-Stage Exchange Arthroplasty for Total Knee Arthroplasty Periprosthetic Joint Infections. J Arthroplasty.

[60] Nabet A, Sax OC, Shanoada R, Conway JD, Mont MA, Delanois RE, Nace J (2022). Survival and Outcomes of 1.5-Stage vs 2-Stage Exchange Total Knee Arthroplasty Following Prosthetic Joint Infection. J Arthroplasty.

[61] Tsung JD, Rohrsheim JA, Whitehouse SL, Wilson MJ, Howell JR (2014). Management of periprosthetic joint infection after total hip arthroplasty using a custom made articulating spacer (CUMARS); the Exeter experience. J Arthroplasty.

[62] Quayle J, Barakat A, Klasan A, Mittal A, Chan G, Gibbs J, Edmondson M, Stott P (2021). Management of peri-prosthetic joint infection and severe bone loss after total hip arthroplasty using a long-stemmed cemented custom-made articulating spacer (CUMARS). BMC Musculoskelet Disord.

[63] Quayle J, Barakat A, Klasan A, Mittal A, Stott P (2022). External validation study of hip peri-prosthetic joint infection with cemented custom-made articulating spacer (CUMARS). Hip Int.

[64] Burastero G, Basso M, Carrega G, Cavagnaro L, Chiarlone F, Salomone C, Papa G, Felli L (2017). Acetabular spacers in 2-stage hip revision: is it worth it? A single-centre retrospective study. Hip Int.

[65] Zhang W, Fang X, Shi T, Cai Y, Huang Z, Zhang C, Lin J, Li W (2020). Cemented prosthesis as spacer for two-stage revision of infected hip prostheses: a similar infection remission rate and a lower complication rate. Bone Joint Res.

[66] Balato M, Petrarca C, de Matteo V, Lenzi M, Festa E, Sellitto A, Campi J, Zarrelli M, Balato G (2021). On the Necessity of a Customized Knee Spacer in Peri-Prosthetic Joint Infection Treatment: 3D Numerical Simulation Results. J Pers Med.

[67] Kong L, Mei J, Ge W, Jin X, Chen X, Zhang X, Zhu C (2021). Application of 3D Printing-Assisted Articulating Spacer in Two-Stage Revision Surgery for Periprosthetic Infection after Total Knee Arthroplasty: A Retrospective Observational Study. Biomed Res Int.

[68] Tsai CH, Hsu HC, Chen HY, Fong YC, Ho MW, Chou CH, Chen YW, Shie MY, Lin TL (2019). A preliminary study of the novel antibiotic-loaded cement computer-aided design-articulating spacer for the treatment of periprosthetic knee infection. J Orthop Surg Res.

[69] Kim TWB, Lopez OJ, Sharkey JP, Marden KR, Murshed MR, Ranganathan SI (2017). 3D printed liner for treatment of periprosthetic joint infections. Med Hypotheses.

[70] Rogers BA, Kuchinad R, Garbedian S, Backstein D, Gross AE, Safir OA (2015). Cement augmentation of the acetabulum for revision total hip arthroplasty for infection. J Arthroplasty.

[71] Um SH, Min BW, Lee KJ, Kim DW, Bae KC, Cho CH, Son ES (2022). Screw augmented cement spacer for deficient acetabulum in periprosthetic infection following Total Hip Arthroplasty. Orthop Traumatol Surg Res.

[72] Rodriguez-Merchan EC, Gomez-Cardero P, Encinas-Ullan CA (2021). Management of bone loss in revision total knee arthroplasty: therapeutic options and results. EFORT Open Rev.

[73] Veltman ES, Moojen DJ, Glehr M, Poolman RW (2016). Similar rate of infection eradication for functional articulating, prefabricated and custom-made spacers in 2-stage revision of the infected total hip: a literature review. Hip Int.

[74] Yamamoto K, Miyagawa N, Masaoka T, Katori Y, Shishido T, Imakiire A (2003). Clinical effectiveness of antibiotic-impregnated cement spacers for the treatment of infected implants of the hip joint. J Orthop Sci.

[75] Fehring TK, Odum S, Calton TF, Mason JB (2000). Articulating versus static spacers in revision total knee arthroplasty for sepsis. The Ranawat Award. Clin Orthop Relat Res.

[76] Emerson RH, Muncie M, Tarbox TR, Higgins LL (2002). Comparison of a static with a mobile spacer in total knee infection. Clin Orthop Relat Res.

[77] Nahhas CR, Chalmers PN, Parvizi J, Sporer SM, Deirmengian GK, Chen AF, Culvern CN, Moric M, Della Valle CJ (2021). Randomized Trial of Static and Articulating Spacers for Treatment of the Infected Total Hip Arthroplasty. J Arthroplasty.

[78] Voleti PB, Baldwin KD, Lee GC (2013). Use of static or articulating spacers for infection following total knee arthroplasty: a systematic literature review. J Bone Joint Surg Am.

[79] Pivec R, Naziri Q, Issa K, Banerjee S, Mont MA (2014). Systematic review comparing static and articulating spacers used for revision of infected total knee arthroplasty. J Arthroplasty.

[80] Guild GN, Wu B, Scuderi GR (2014). Articulating vs. Static antibiotic impregnated spacers in revision total knee arthroplasty for sepsis. A systematic review. J Arthroplasty.

[81] Johnson AJ, Sayeed SA, Naziri Q, Khanuja HS, Mont MA (2012). Minimizing dynamic knee spacer complications in infected revision arthroplasty. Clin Orthop Relat Res.

[82] Sporer SM (2020). Spacer Design Options and Consideration for Periprosthetic Joint Infection. J Arthroplasty.

[83] DeBoer DK (2020). Comparison of Traditional Molded, First-Generation Premolded, and Second-Generation Premolded Antibiotic-Loaded Polymethylmethacrylate Articulating Spacers for Treatment of Chronic Prosthetic Joint Infection of the Knee. J Arthroplasty.

[84] Spinarelli A, Bizzoca D, Moretti L, Vicenti G, Garofalo R, Moretti B (2022). The autoclaving and re-implantation of an infected prosthesis as a spacer during resection knee arthroplasty: a systematic review. Musculoskelet Surg.

[85] Kugelman D, Roof M, Egol A, Guanche I, Chen AF, Schwarzkopf R, Aggarwal VK (2022). Comparing Articulating Spacers for Periprosthetic Joint Infection After Primary Total Hip Arthroplasty: All-Cement Versus Real-Component Articulating Spacers. J Arthroplasty.

[86] Yu Q, Luo M, Wu S, Lai A, Sun Y, Hu Q, He Y, Tian J (2019). Comparison of infection eradication rate of using articulating spacers containing bio-inert materials versus all-cement articulating spacers in revision of infected TKA: a systematic review and meta-analysis. Arch Orthop Trauma Surg.

[87] Spivey JC, Guild GN, Scuderi GR (2017). Use of Articulating Spacer Technique in Revision Total Knee Arthroplasty Complicated by Sepsis: A Systematic Meta-Analysis. Orthopedics.

[88] Grosso MJ, Kozaily E, Cacciola G, Parvizi J (2021). Characterizing Femoral and Acetabular Bone Loss in Two-Stage Revision Total Hip Arthroplasty for Infection. J Arthroplasty.

